# Wetting transitions in droplet drying on soft materials

**DOI:** 10.1038/s41467-019-12093-w

**Published:** 2019-10-21

**Authors:** Julia Gerber, Tobias Lendenmann, Hadi Eghlidi, Thomas M. Schutzius, Dimos Poulikakos

**Affiliations:** Laboratory of Thermodynamics in Emerging Technologies, Department of Mechanical and Process Engineering, ETH Zurich, Sonneggstrasse 3, CH-8092 Zurich, Switzerland

**Keywords:** Soft materials, Wetting, Applied physics, Fluid dynamics

## Abstract

Droplet interactions with compliant materials are familiar, but surprisingly complex processes of importance to the manufacturing, chemical, and garment industries. Despite progress—previous research indicates that mesoscopic substrate deformations can enhance droplet drying or slow down spreading dynamics—our understanding of how the intertwined effects of transient wetting phenomena and substrate deformation affect drying remains incomplete. Here we show that above a critical receding contact line speed during drying, a previously not observed wetting transition occurs. We employ 4D confocal reference-free traction force microscopy (cTFM) to quantify the transient displacement and stress fields with the needed resolution, revealing high and asymmetric local substrate deformations leading to contact line pinning, illustrating a rate-dependent wettability on viscoelastic solids. Our study has significance for understanding the liquid removal mechanism on compliant substrates and for the associated surface design considerations. The developed methodology paves the way to study complex dynamic compliant substrate phenomena.

## Abstract (web summary)

It has been shown previously that substrate viscoelasticity affects surface wettability. Here the authors observe a wetting transition during drying of droplets on such substrates and elucidate it with high resolution force field measurements thereby determining its dependence on substrate properties.

## Introduction

Surface engineering of rigid materials to passively control water droplet wetting behavior has gained a great deal of attention due to the unique attributes of these surfaces such as self-cleaning^1^, liquid and ice repellency^2–7^, and water collection enhancement^8,9^. For practical applications, understanding how droplets adhere, slide, and remove themselves from surfaces (including shedding, jumping, and drying) is of great fundamental importance, and numerous studies have investigated the physics of droplet motion and removal from rigid substrates and the diverse set of behavior for a range of liquid properties^10,11^ and environmental conditions^12–14^. However, while the dynamic wetting behavior of droplets on rigid materials is relatively well-understood, reports on the more complex and very important dynamics of droplet removal from compliant materials is comparatively scant^15–21^. Notable works include Pu et al.^19^ who investigated the effect of substrate viscoelasticity on the wetting and drying behavior of droplets and Lopes et al.^20^ who showed that deformation near the contact line influences evaporation of a sessile droplet. Other work has shown that compliance can fundamentally alter droplet–substrate and droplet–droplet interactions, control droplet spreading^22–24^, enhance superhydrophobicity and icephobicity^7,25^, enable collective droplet motion^26,27^, and enhance water collection^28,29^. However, the physics of the receding contact line during evaporation of droplets at the mesoscale—critical to understanding and controlling the removal process—is not well-understood. Although the driving mechanisms are controlled by phenomena at the micro- and nanoscales, existing studies are limited to macroscopic characterization^20^ while the theoretical work often uses simplifying assumptions, such as linear elasticity, equilibrium, constant contact line velocity or symmetric surface energies/stresses^22,23,30–34^. To understand droplet drying and removal from compliant substrates, we need high-resolution, transient information of the intertwined phenomena of mesoscopic substrate deformation and wetting behavior.

Here, we investigate the mutual influence of substrate viscoelasticity and associated transient mesoscopic substrate deformations on droplet receding dynamics during drying for a range of environmental conditions and material compositions regulating the process. For this purpose, we have advanced and tailored a high-speed four-dimensional confocal reference-free traction force microscopy (4D cTFM) method^35^ (three spatial dimensions and time). We observe that when the contact line velocity approaches and exceeds a characteristic substrate relaxation rate, then the apparent receding contact angle significantly decreases, undergoing a wetting transition. During drying, we found that the wetting ridge is initially symmetric with capillarity driving a decrease in the droplet–substrate contact radius. Then, as evaporation proceeds, viscoelastic effects become important and resist a further decrease in contact radius, which manifest themselves as a surge in substrate deformation and wetting ridge “stretching”. This signifies that the wetting ridge acts as a viscoelastic brake, retarding the droplet receding motion. A decrease in the apparent contact angle is to be understood in conjunction with the sharp inward bent of the wetting ridge apex, which acts to pin the contact line and gives rise to a wetting transition. These findings are expected to have importance in a broad range of fields, impacting liquid retention/removal from compliant substrates and resolution of 3D printing of soft materials.

## Results

### Droplets drying on rigid and compliant substrates

In the experiments, a single water droplet (*V*_placed_ = 71.9 ± 8.7 nL) was placed on a poly(dimethylsiloxane) (PDMS Sylgard 184, in the following abbreviated as PDMS) coated glass substrate, which was located inside an environmental chamber. The thickness of the coating was 30 μm. We tuned the stiffness of the film by varying the mixing ratio of the prepolymer and catalyst. To tune the droplet evaporation rate, we changed the relative humidity (*rH* = 15, 50, and 90%) in the environmental chamber while keeping the temperature constant (*T* = 24.1 ± 0.5 °C). Care was taken to obtain droplets of practically equal volume when placed on the substrate; however, the start of the measurement, *t*_0_, was set to be the time when the droplet reached a volume of 55 nL. The initial contact angle was found to be different depending on sample stiffness; $\theta _0^ \ast = 112.7 \pm 2.2^\circ$ on the rigid samples (PDMS, 9:1) versus $\theta _0^ \ast = 119.6 \pm 2.5^\circ$ on the compliant samples (PDMS, 50:1), and the corresponding initial contact radii were *R*_0_ = 258.6 ± 11.9 μm and *R*_0_ = 241.4 ± 15.3 μm, respectively. We observed the wetting behavior of the evaporating droplets with a side-view camera tilted ~5° with respect to horizontal.

**Fig. 1:**
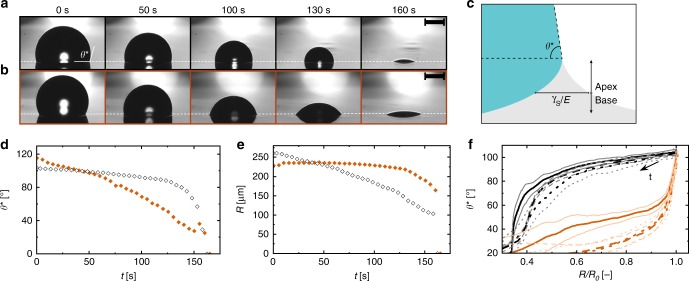
Sessile droplets drying on rigid and compliant substrates. Image sequence of sessile droplets evaporating from, **a** rigid (PDMS Sylgard 184 9:1, *E* = 2937 kPa) and, **b** compliant (bottom-row, PDMS Sylgard 184 50:1, *E* = 20 kPa^20^) substrates (environmental conditions: *rH* = 50% and *T* = 24.1 ± 0.5 °C). Location of the temperature measurement, *T*, is shown in Fig. 2a. **c** Schematic of a droplet on a compliant substrate and resulting deformation. We defined the apex region as the portion of the wetting ridge which has a width less than *Υ*_S_/*E*. **d** Experimentally measured apparent contact angle, θ^*^, vs. time, *t*, of the droplets in (**a**) and (**b**) (empty diamonds: rigid; filled diamonds: compliant). **e** Experimentally measured contact radius, *R*, vs. *t* of the droplets in (**a**) and (**b**) (empty diamonds: rigid; filled diamonds: compliant). **f** θ^*^ vs. normalized contact radius, *R/R*_0_, where *R*_0_ is the value of *R* at *t*_0_, for *rH* values of 15 ± 1% (dotted line), 50 ± 1% (dashed line), and 90 ± 1% (solid line) on rigid (black) and compliant (red) substrates. Each line corresponds to the average of nine independent measurements; fine lines represent the standard deviations. Scale bar: **a**, **b** 200 µm. Source data are provided as a Source Data file for (**d**–**f**)

Figure 1a, b shows image sequences of water droplets drying on substrates with two different values of the Young’s modulus, *E* (Fig. 1a: *E* = 2937 kPa, rigid; Fig. 1b: *E* = 20 kPa, compliant), in an environment at *T* = 24.1 °C and *rH* = 50%. It is clear that while the time it takes for a droplet to evaporate is similar in these two cases, the values of the apparent contact angle, θ^*^, as shown in Fig. 1a, b, greatly differ, even though both samples have the same chemical composition. They are both made of polydimethylsiloxane, just with a varying crosslinking ratio. Figure 1c shows a schematic of the wetting of a droplet on a compliant substrate, defining θ^*^ for a compliant substrate and the wetting ridge apex and base. The horizontal scale of the wetting ridge apex is defined as the region where nonlinear elastic behavior is expected, $\Upsilon _{\mathrm{S}}/E$^32,36,37^, with $\Upsilon _{\mathrm{S}}$ being the solid surface tension, which under the assumption that solid–vapor and solid–liquid surface tension are similar, can be approximated by the average of the two surface tensions. Figure 1d shows a plot of θ^*^ vs. time, *t*, for the cases in Fig. 1a, b. We see that for the droplet drying on a rigid substrate, θ^*^ is approximately constant until the droplet becomes small and then θ^*^ decreases rapidly. On the other hand, for the droplet drying on the compliant substrate, θ^* ^decreases steadily. Figure 1e shows a plot of droplet contact radius, *R*, vs. *t* for the cases in Fig. 1a, b. On the rigid substrate *R* decreases faster than on the compliant substrate. Thus, during most of the evaporation process the contact line velocity is smaller on the compliant substrate than on the rigid one (except for the last ~10 s of vaporization). Figure 1f shows a plot of θ^*^ vs. *R*/*R*_0_ for two different values of *E* and three different *rH* values (subscript “0” denotes value at *t*_0_). We see that for rigid substrates, *rH*—and therefore the evaporation rate—has no significant influence on the θ^*^ dependence of radial position. Droplets evaporating on rigid substrates always show the same trend of a longer period of relatively constant θ^*^ followed by a pinning of the contact line and a rapid decrease in θ^*^ toward the end of the evaporation. On the other hand, for the compliant substrates, we see that *rH* has a significant impact on θ^*^. For *rH* values of 15 and 50%, the drying behavior is similar. The contact line appears to be pinned for large droplets; therefore, *R* stays nearly constant while θ^*^ decreases. Then, when the droplet is relatively small, *R* decreases significantly while θ^*^only changes moderately. In contrast, for a relative humidity of 90%, the transition from constant contact radius toward a moving contact line occurs at a considerably larger value of θ^*^ (~70° instead of ~40°). Therefore, we see that both *rH* and *E* have a significant impact on θ^*^, giving rise to a wetting transition from high values of θ^*^ to low values of θ^*^ at low *rH*.

Various works indicate a dependence of substrate deformation on compliant film thickness^34,36,38^; therefore, we have investigated the effect of film thickness on the drying behavior (see Supplementary Fig. 1). We did not observe any significant influence of film thicknesses between 15 and 220 µm on the wetting behavior. Hence, it can be assumed that these film thicknesses are in the semifinite regime, and that the as-formed wetting ridges are similar in the vicinity of the contact line, because it is the orientation of the interfaces within the region of the wetting ridge apex that determines the apparent contact angle. We aim to elucidate the observed wetting transition by observing simultaneously the transient mesoscopic substrate deformations near the droplet contact line and the macroscopic wetting behavior.

### Transient deformation field detection during droplet drying

We extended and employed a 4D cTFM method, which was introduced in an earlier publication^35^. The present technique is able to characterize the transient surface deformation and stress vector fields during droplet evaporation, while simultaneously monitoring the macroscopic wetting behavior. In contrast to confocal traction force microscopy, where the original methodology is applied^35,39^, the time scales are much shorter and the magnitude of displacements are considerably larger in our case, meaning that we added the capability to resolve large displacements in-plane and out-of-plane with high temporal resolution (see Methods and Supplementary Note 1: Reference-free cTFM Measurement). Our ultimate goal is to relate the observed mesoscopic surface deformation fields to the macroscopic wetting behavior. In these experiments, the compliant substrate is overlaid with a regular triangular arranged array of red quantum dot (QD) discs, each containing a countable number of QDs, fabricated with an electrohydrodynamic inkjet printing process (NanoDrip) we developed earlier (see Methods)^40,41^. We image the transient mesoscopic surface deformations at the moving contact line region with fluorescence microscopy and quantify their position in four dimensions.

**Fig. 2:**
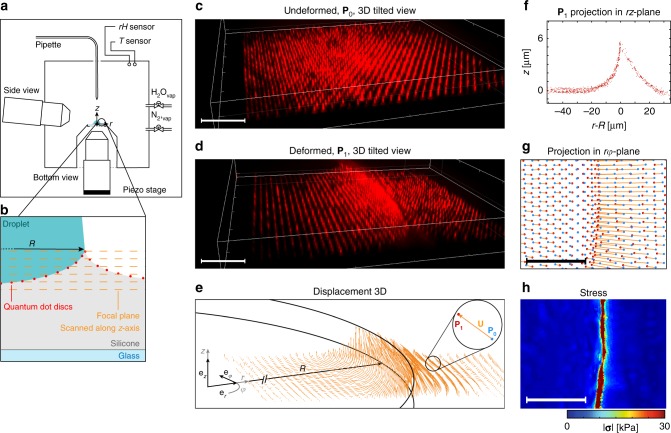
Detection of transient mesoscale deformation fields during droplet drying. **a** Schematic of the setup used in all experiments. It consists of an environmental chamber, where *rH* and *T* are controlled, two objectives (bottom and side views), and a pipette for dispensing droplets. The bottom objective is attached to a piezo stage. **b** Schematic showing a droplet on a compliant silicone substrate, creating a wetting ridge at its contact line. The surface is patterned with a regular array of quantum dot discs, which are detected by a custom-built bright-field fluorescence microscope. By scanning in the *z*-direction, we can determine surface deformations in this direction. Tilted-view fluorescence micrographs of compliant substrates with a triangular array of red QD discs in the **c** unloaded (no droplet) and **d** loaded state (with droplet). Through microscopy, we can detect the placement field of the loaded state, **P**_1_, and by a relaxation algorithm we can find the unloaded state, **P**_0_ (see Methods, Supplementary Note 1: Reference-free cTFM Measurement and Supplementary Fig. 2). Then the displacement field is defined as **U** = **P**_1_ − **P**_0_. In **d**, we focused only on a small portion of the substrate and contact line in order to reveal the significant substrate 3D deformations. **e** 3D displacement field, **U**, for the deformed substrate shown in (**d**). The displacement vector is defined as, **U** = *U*_*r*_**e**_*r*_ + *U*_*φ*_**e**_*φ*_ + *U*_*z*_**e**_*z*_, and each vector is represented by an arrow in (**e**). Also shown in (**e**) is the cylindrical coordinate system; the origin is at the center of the droplet−substrate interface; the black circles represent constant values of *r* and constant *z* lines at *r* = *R* and *z* = 0 and *z* = 6 μm. **f** Projection in the *rz*-plane of all **P**_1_ of the deformed substrate data shown in (**d**). **g** Magnified projection of **U** (from **e**) in the *rφ*-plane. In-plane positions (orange lines) of **P**_0_ (blue dots) to **P**_1_ (red dots). **h** Magnified stress vector field magnitude of the deformed substrate shown in (**d**) computed by finite element analysis. Scale bars: **c**, **d**, 20 µm **g**, **h**, 20 µm. Source data are provided as a Source Data file for (**c**–**f**)

Figure 2a shows the experimental setup consisting of an environmental chamber in which the QD disc patterned substrate is placed upon an elevated sample holder. The chamber contains a side-view camera, inlets for nitrogen gas and water vapor, a pipette, a relative humidity sensor, and temperature sensors. The environmental chamber is placed on top of an inverted home-built bright-field fluorescence microscope, with its bottom objective mounted on a piezo stage allowing the rapid shifting of the focal plane in the *z*-direction. The schematic in Fig. 2b shows a droplet, the silicone coating, QD discs, and the glass substrate. It also shows how we scanned the focal plane along the *z*-axis to measure the QD disc *z* positions. With the side-view camera, we measured θ^*^ and *R*, and with this we were able to compute the contact line velocity (*dR*/*dt*). The bottom-view allowed us to acquire the mesoscopic surface deformations. Figure 2c, d shows 3D images of an undeformed and deformed QD disc patterned substrate (*E* = 12.6 kPa), resepectively, from a tilted-view perspective. The deformation is due to contact with a water droplet (*rH* = 15%). Note that the QD discs are fixed on the surface, and their motion accurately tracks the motion of the compliant surface. Therefore, the positions of the QD discs in Fig. 2c, d form the placement fields **P**_0_ and **P**_1_, respectively. Figure 2e shows the resulting 3D displacement field, defined as **U** = **P**_1_ − **P**_0_, for the case in Fig. 2d along with the droplet radius and the cylindrical coordinate system used here. Since the substrate consists of a regular array of QD discs, the relative positions of **P**_0_ within the array are known a priori, making the present technique reference-free (we do not need to acquire an image of the substrate in the unloaded state). In short, the QD discs are deposited in an array that is well-defined; the droplet is placed in contact with the surface and substrate deformations are observed; by using the edges of the array as a boundary condition, we can determine the absolute displacements of all QD discs on the surface. The original algorithm used in ref. ^35^ had to be markedly expanded for our purposes (see Methods, Supplementary Note 1: Reference-free cTFM Measurement and Supplementary Fig. 2). We can represent the displacement field in cylindrical coordinates as, ${\mathbf{U}} = U_r {\mathbf{e}}_r + U_\varphi {\mathbf{e}}_\varphi + U_z {\mathbf{e}}_z$, which has its origin at the center of the droplet, where **e**_*r*_, **e**_*φ*_, and **e**_*z*_ are the unit vectors in *r*, *φ*, and *z* and *U*_*r*_, *U*_*φ*_, and *U*_*z*_ are the radial, azimuthal, and vertical displacements, respectively. Figure 2f is a projection of the placement field **P**_1_ in the *rz*-plane. Since the droplet is axisymmetric, this allows us to have a clearer picture of the wetting ridge shape. Figure 2g plots a projection of **P**_0_, **P**_1_, and **U** in the *rφ*-plane. By knowing **U** and using the constitutive material relations (see Methods), we are able to compute the stress vector field across the substrate surface, **σ**(**n**), with the normal vector to the deformed surface, **n** (ref. ^35^). Figure 2h shows the associated magnitude of the stress vector field, |**σ**(**n**)|, computed with finite element analysis (FEA).

### The effect of humidity and compliance on droplet drying

**Fig. 3:**
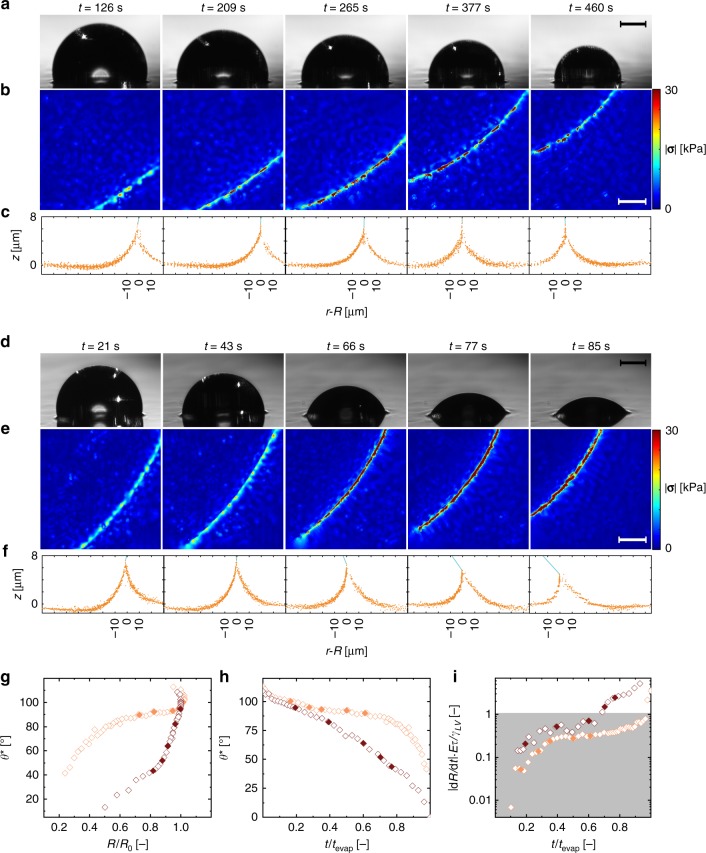
Characterizing the effects of humidity and compliance on droplet de-wetting with 4D confocal reference-free traction force microscopy. **a** Time series of the macroscopic side-view showing a droplet slowly evaporating (*rH* = 90%, *T* = 24 °C, *V*_placed_ = 67 nL) from a compliant solid (Silicone CY 52–276 9:10), with **b** simultaneous stress vector field magnitude at the contact line and, **c** simultaneous mesoscopic shape of the wetting ridge. Blue lines represent liquid–vapor interface. **d** Time series of the macroscopic side-view showing a droplet quickly evaporating (*rH* = 15%, *T* = 24 °C, *V*_placed_ = 49 nL) from a compliant solid (Silicone CY 52–276 9:10), with **e** simultaneous stress vector field magnitude at the contact line and **f** simultaneous mesoscopic shape of the wetting ridge. Blue lines represent liquid–vapor interface. **g** Plot of θ^*^ vs. *R*/*R*_0_ of the droplets in (**a**) (orange) and (**d**) (red). The filled diamonds represent the time steps shown in (**a**) and (**d**). **h** θ^*^ vs. *t*/*t*_evap_ for the droplets in (**a**) (orange) and (**d**) (red). **i** Absolute normalized contact line speed, |*dR*/*dt*|*Eτ*/*γ*_LV_, vs. *t*/*t*_evap_ for the droplets in (**a**) (orange) and (**d**) (red). Scale bars: **a**, **d**, 150 µm, **b**, **e**, 20 µm. Source data are provided as a Source Data file for (**c**, **f**–**i**)

Figure 3a, d shows side-view image sequences of two droplets evaporating on a compliant substrate (Silicone CY 52–276, in the following abbreviated as Silicone CY, in the ratio A:B of 9:10, *E* = 12.6 kPa) in an environment with *rH* values of 90% (slow evaporation) and 15% (fast evaporation), respectively (see Supplementary Movie 1, see also Supplementary Fig. 3 for the same experiment on a different compliant material). Also shown are the synchronous substrate stress vector magnitudes (Fig. 3b, e) and the wetting ridge shapes that we measured (Fig. 3c, f, projection of **P**_1_ in *rz*-plane). Both cases capture the transition from a static to moving receding contact line. Figure 3c, f reveals that the wetting ridge shape is not always symmetric with respect to the *z*-axis at *r* = *R *in the *rz*-plane. The wetting ridge shape during fast evaporation, shown in Fig. 3f, is markedly tilted toward the droplet, whereas during the slow evaporation, shown in Fig. 3c, the wetting ridge is relatively symmetric with respect to the *z*-axis. Figure 3g, h shows plots of θ^*^ vs. *R*/*R*_0_ and θ^*^ vs. *t*/*t*_evap_, respectively, for two different environmental conditions, *rH* = 15 and 90%. Here, *t*_evap_ is the time it takes for a droplet to evaporate. Figure 3i shows a plot of *dR/dt* vs. *t*, where *dR/dt* is normalized by a characteristic rate of substrate relaxation, which we define next. The characteristic rate of substrate relaxation is droplet–substrate dependent and is defined as the length scale of substrate deformation divided by the relaxation time of the substrate, *τ* (characteristic substrate relaxation time). We determined *τ* experimentally by performing step-strain tests and fitting a generalized Maxwell solid model with two elements to the data (see Methods and Supplementary Figs. 4–9 for measurements of *τ* and *E* for the materials used in this study). The typical length scale of substrate deformation was defined according to the literature^30,36^ as *γ*_LV_/*E*, where *γ*_LV_ is the liquid surface tension. Substituting appropriate values yields *γ*_LV_/(*Eτ*) = 0.98 µm s^−1^ (*γ*_LV_ = 71.97 mN m^−1^, *E* = 12.6 kPa, *τ* = 5.8 s). During rapid evaporation, *dR*/*dt* becomes comparable in magnitude to, and at late times, even exceeds *γ*_LV_/(*Eτ*). At these times, we also observe relatively large surface stresses at the contact line and asymmetric wetting ridges. This characteristic rate of substrate relaxation also appears in the context of droplet spreading on soft substrates^42,43^. (See Supplementary Note 2: Considerations for Appropriate Material Model Selection and Supplementary Fig. 10 for rheology measurements of PDMS 50:1 and Silicone CY 9:10, which do not exhibit viscous-to-elastic transition points and therefore cannot be used to determine a cross-over frequency between the storage and loss moduli.)

Again, it is clear that *rH* has significant influence over the droplet wetting behavior when drying on a compliant substrate and that the average of |**σ**(**n**)| at the contact line is increasing with time for both cases. Furthermore, the values of |**σ**(**n**)| measured during fast evaporation exceed those during slow evaporation. Due to the fact that the magnitude of the inherent surface tension pulling on the substrate, *γ*_LV_, remains constant—only its direction changes according to the instantaneous contact angle—the increase in measured |**σ**(**n**)| indicates an accumulation of stress that cannot be dissipated on such short time scales. We observe that this increase in substrate stress occurs concurrently with a significant change in the wetting ridge shape (as seen in Fig. 3e, f from *t* = 66 s to *t* = 85). At equilibrium, the wetting ridge can bend if *γ*_SL_ ≠ *γ*_SV_^44^; however, we are investigating dynamic phenomena and we have no expectation of equilibrium or fulfillment of Neumann’s law (see Supplementary Note 3: Force Balance at Contact Line and Strain Dependence Considerations and Supplementary Fig. 11). Here, the wetting ridge shape and tilt are controlled by the interplay of wetting dynamics and substrate viscoelasticity near the contact line.

### Wetting transitions in droplet drying on compliant materials

**Fig. 4:**
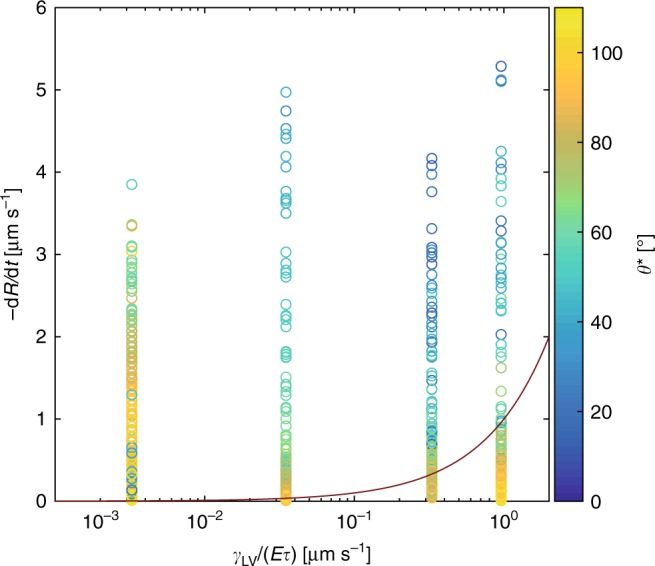
Wetting transition phase diagram for droplet drying on soft materials. Contact line velocity, *dR/dt*, vs. the characteristic rate of substrate relaxation, *γ*_LV_/(*Eτ*), vs. the apparent contact angle, θ^*^.This plot contains droplet evaporation experiments on four materials, PDMS Sylgard 184 9:1 (*γ*_LV_/(*Eτ*) = 3.3 × 10^−^^3^ µm s^−1^), PDMS Sylgard 184 30:1 (*γ*_LV_/(*Eτ*) = 3.5 × 10^−2^ µm s^−1^), Silicone CY 52–276 5:6 (*γ*_LV_/(*Eτ*) = 0.33 µm s^−^^1^), and Silicone CY 52–276 9:10 (*γ*_LV_/(*Eτ*) = 0.98 µm s^−^^1^), and under varying relative humidity (in the range *rH* = 0% to *rH* = 95%), at room temperature (*T* = 24 °C). In total, there are *N* = 44 individual drying experiments. The red line marks the relation when −*dR*/*dt* = *γ*_LV_/(*Eτ*). Source data are provided as a Source Data file

To test whether the proposed wetting transition criterion holds for other materials, we ran experiments where we varied substrate stiffness and relative humidity. Figure 4 shows a plot of receding velocity, *−dR/dt*, vs. $\gamma _{{\mathrm{LV}}}/(E\tau )$ for four different materials. For the hardest material (PDMS 9:1, *E* = 2937 kPa) there is no clear transition toward lower contact angles depending on the contact line velocity. This can be explained through the fact that the substrate deformations are almost negligible $(\gamma _{{\mathrm{LV}}}/E \approx 24\,{\mathrm{nm}})$ and the contact line velocity is not expected to be influenced by the dissipation within the solid. Rather, the shape of the droplet during receding is solely dominated by liquid surface tension (*Ca* = *η*|*dR*/*dt|*/*γ*_LV_ < 10^−7^, with *η* being the liquid viscosity). For the other three materials, however, we can observe a transition toward lower contact angles with increasing |*dR/dt*| and this transition happens at higher |*dR/dt*| for higher values of $\gamma _{{\mathrm{LV}}}/(E\tau )$. Also shown is the condition where $- dR/dt = \gamma _{{\mathrm{LV}}}/(E\tau )$ and this line coincides well with the actual observed transition toward lower contact angles.

### Mechanism of the dynamically triggered wetting transition

**Fig. 5:**
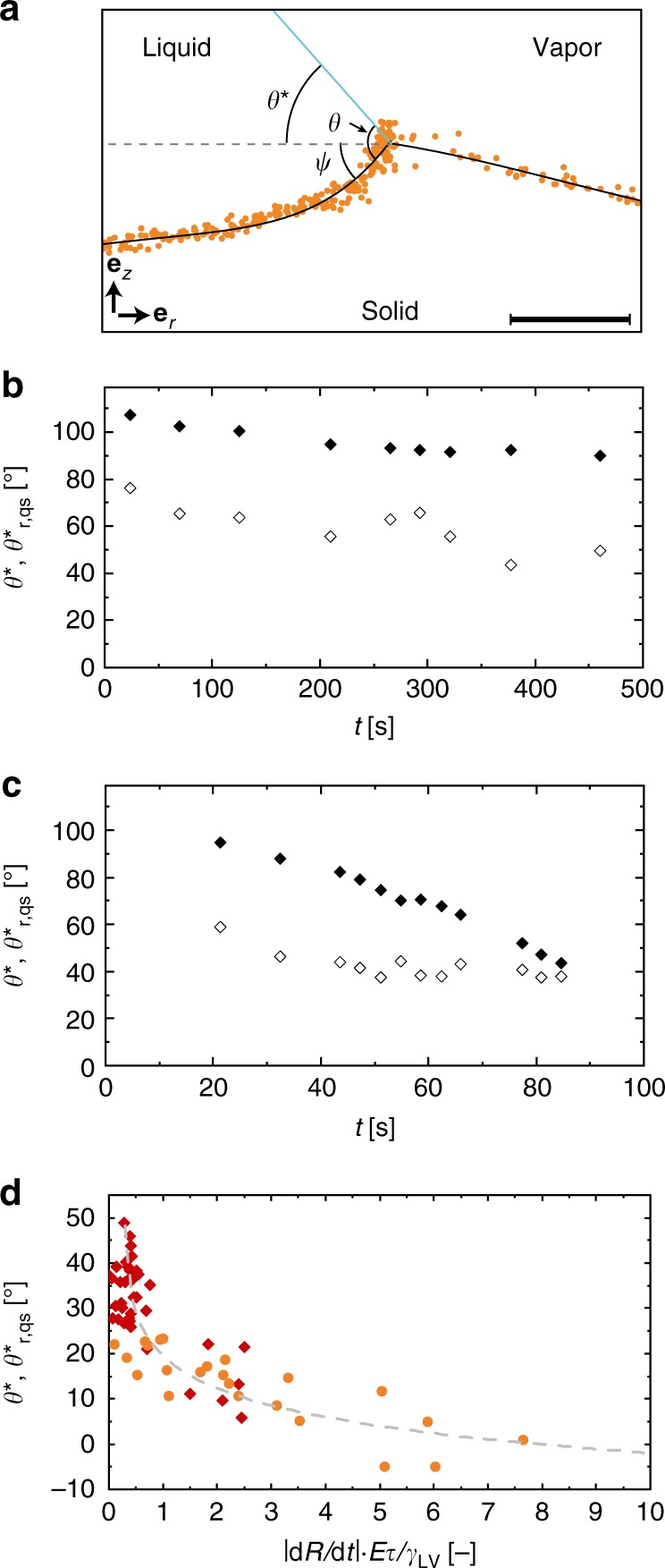
Mechanism of dynamically triggered wetting transition. **a** Schematic showing a contact line pinned on a sharp quasi-static apex and the definitions of the apparent contact angle, θ^*^, the angle between the liquid–solid interface and the horizontal, *ψ*, and the angle between the liquid–vapor and liquid–solid interface, *θ*. At contact line depinning, the condition *θ* = *θ*_r_ has to be satisfied. The black line is a piecewise polynomial fit of fifth order. Theoretical receding contact angle, $\theta _{{{\mathrm{r,qs}}}}^ \ast$ (empty diamonds), and θ^*^ (filled diamonds) vs. *t* for a contact line pinned on a quasi-static apex for **b** slow evaporation (*rH* = 90%, droplet from Fig. 3a) and **c** rapid evaporation (*rH* = 15%, droplet from Fig. 3d) of a droplet on a compliant substrate. **d** Difference between the apparent contact angle, θ^*^, and the theoretical receding contact angle if the wetting ridge acts as a quasi-static defect, $\theta _{{{\mathrm{r,qs}}}}^ \ast$, as a function of |*dR*/*dt*|*Eτ*/*γ*_LV_. We tested two material compositions of Silicone CY 52–276, ratio 5:6 (orange, *N* = 4, with 24 time steps) and ratio 9:10 (red, *N* = 5, with 38 time steps), with *rH* of 15 ± 1% and 90 ± 1% and at room temperature (*T* = 24 °C). The gray dashed line is a logarithmic fit with three parameters. Scale bar: **a** 5 µm (with an aspect ratio of 1:1). Source data are provided as a Source Data file for (**b**–**d**)

We are interested in understanding this dynamically triggered wetting transition, which occurs during contact line receding; therefore, our interest is in the observed receding contact angle related to the concurrent substrate response. Figure 5a shows an experiment where a water droplet is on a compliant substrate drying quickly. The droplet shape (liquid–vapor interface) is determined experimentally and is given by the blue line. Also shown are the projected values of **P**_1_ in the *rz*-plane. With a best fit line (black line), we can then construct the shape of the liquid–solid and solid–vapor interfaces, assuming that the shape of the wetting ridge is axisymmetric. With this we can construct the shapes of the liquid–vapor, solid–liquid, and solid–vapor interfaces near the contact line. At a given point along the contact line, we define the angles of the liquid–vapor and liquid–solid interfaces with respect to the horizontal as θ^*^ and *ψ*, respectively. The angle between the liquid–vapor and liquid–solid interfaces we define as *θ*.

To understand the observed wetting behavior, it is instructive to consider the case where the droplet contact line moves much faster than the wetting ridge can reorganize, and the substrate is, therefore, in a quasi-static state. Therefore, in order for the droplet to depin from the apex, the following criterion must be satisfied: *θ* = *θ*_r_, with *θ*_r_ being the receding contact angle. At the point just before depinning, and assuming a quasi-static wetting ridge, we can define the apparent receding contact angle, $\theta _{{{\mathrm{r,qs}}}}^ \ast = \theta _{\mathrm{r}} - \psi$, where the subscript “qs” denotes the quasi-static-wetting-ridge assumption^45^. Here, *ψ* is found experimentally (see above). *θ*_r_ is also found experimentally by measuring its value on a smooth hard substrate with similar composition to the compliant substrate (rigid smooth PDMS substrate: *E* = 2937 kPa, *θ*_r_ = 99.7 ± 0.5°). Figure 5b, c shows plots of $\theta _{{\mathrm{r,qs}}}^ \ast$ (theoretical) and θ^*^ (experimental) vs. *t* for the high and low *rH* cases from Fig. 3a, d, respectively. We observed that $\theta _{{\mathrm{r,qs}}}^ \ast$ is always smaller than θ^*^, and that for the fast evaporating droplet, especially for late times when θ^*^ is at its minimum, $\theta _{{\mathrm{r,qs}}}^ \ast$ is in excellent agreement with θ^*^. Therefore, for fast receding dynamics, the assumption of a quasi-static wetting ridge holds and facilitates the understanding of the observed wetting transition. Figure 5d shows a plot of $(\theta ^ \ast - \theta _{{\mathrm{r,qs}}}^ \ast )$ vs. |*dR*/*dt*|*Eτ*/*γ*_LV_ for two different compliant substrates, and what we see is that for both cases, there is an inverse correlation that approaches 0° when the receding contact line velocity strongly exceeds *γ*_LV_/(*Eτ*). However, we still never observed a contact line depin from the wetting ridge while the droplet evaporated, which we attributed to the fact that the wetting ridge becomes more asymmetric the faster the contact line moves, making depinning less likely. It is possible that it does happen in our experiments, but the droplets are too small and move too fast to capture this event.

### Displacement and tension in droplet drying on soft materials

**Fig. 6:**
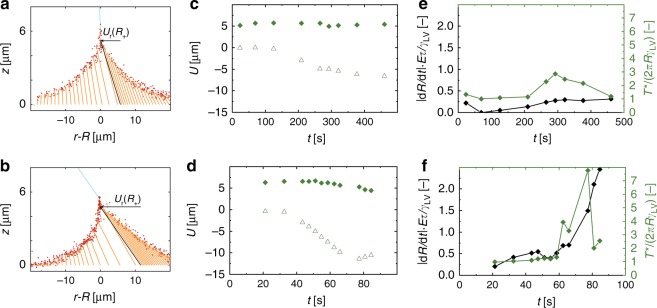
Displacement and effective line tension of a drying droplet on a compliant substrate. **a** Wetting ridge shape and displacement vectors projected to the *rz*-plane at *t* = 460 s during slow evaporation (*rH* = 90%, droplet in Fig. 3a). **b** Wetting ridge shape and displacement vectors projected to the *rz*-plane at *t* = 77 s during fast evaporation (*rH* = 15%, droplet in Fig. 3d). The black vectors in (**a**) and (**b**) mark **U**(*r* = *R*_+_) and the blue lines in (**a**) and (**b**) mark the liquid–vapor interface. Plot of vertical displacement, *U*_*z*_(*r* = *R*) (diamonds), and radial displacement just outside the contact line, *U*_*r*_(*r* = *R*_+_) (triangles), for **c** slow evaporation (*rH* = 90%, droplet from Fig. 3a) and **d** fast evaporation (*rH* = 15%, droplet from Fig. 3d). Plots of |*dR*/*dt*|*Eτ*/*γ*_LV_ vs. normalized effective line tension, T^*^/(2*πRγ*_LV_), vs. *t* for **e** slow evaporation (*rH* = 90%, droplet from Fig. 3a) and **f** fast evaporation (*rH* = 15%, droplet from Fig. 3d). Source data are provided as a Source Data file for (**c**–**f**)

In order to understand the dynamics of the wetting ridge and the validity of the “quasi-static ridge” assumption, we analyzed the individual contributions to the displacement field near the contact line and the total substrate stresses. In Fig. 6a, b, we present the wetting ridge shapes and projections of **U** in the *rz*-plane at one time step during slow evaporation (*rH* = 90%, *t* = 460 s in Fig. 3a) and fast evaporation (*rH* = 15%, *t* = 77 s in Fig. 3d). The radial displacements just outside the contact line, *U*_*r*_(*r* = *R*_+_), are considerably larger during fast evaporation, whereas the vertical displacements at the contact line, *U*_*z*_(*r* = *R*), are almost equal during fast and slow evaporation. Figure 6c, d shows the evolution of *U*_*r*_(*r* = *R*_+_) and *U*_*z*_(*r* = *R*) over the course of evaporation for the high and low-*rH* cases from Fig. 3a, d, respectively. We found that the radial inward pull grew considerably larger during fast evaporation than during slow evaporation, indicating that the wetting ridge base cannot move as fast as the apex during rapid evaporation and the wetting ridge becomes stretched. This stretching is quantified by the significantly higher measured radial displacements associated with the case of faster evaporation.

To quantify the above with respect to droplet surface tension, we integrated the stresses in the vicinity of the contact line to obtain a term for the effective line tension, T^*^ [J m^−1^]

1 $${T^ \ast = \mathop {\int}\limits_A {\left| {{\mathbf{\upsigma }}\left( {\mathbf{n}} \right)} \right|dA} = \mathop {\int}\limits_{R - \Upsilon _{\mathrm{S}}/E}^{R + \Upsilon _{\mathrm{S}}/E} {\mathop {\int}\limits_0^{2\pi } {\left| {{\mathbf{\upsigma }}\left( {\mathbf{n}} \right)} \right|d\varphi dr} } \cong \frac{{2\pi }}{\alpha }\mathop {\int}\limits_{R - \Upsilon _{\mathrm{S}}/E}^{R + \Upsilon _{\mathrm{S}}/E} {\mathop {\int}\limits_0^\alpha {\left| {{\mathbf{\upsigma }}\left( {\mathbf{n}} \right)} \right|d\varphi dr} }} .$$

This term was determined by integrating the magnitude of the stresses, |**σ**(**n**)| [Pa], over an area that is defined by an opening angle, *α* (the region of the contact line that is visible), and from *r* = *R* − *Υ*_S_/*E* to *r* = *R* + *Υ*_S_/*E* (see Methods and Supplementary Fig. 12 for illustration of the integration area). Figure 6e, f plots |*dR*/*dt*|*Eτ*/*γ*_LV_ vs. T^*^/(2*πRγ*_LV_) vs. *t* for *rH* = 90% (slow evaporation, Fig. 3a) and *rH* = 15% (fast evaporation, Fig. 3d), respectively. The effective line tension is a measure of the elastic energy stored in the substrate, ≅2πRT^*^ [J]. For the fast evaporating droplet, the experimentally measured stresses here significantly exceed those expected due to surface tension alone and are much larger than that measured for the slowly evaporating droplet. To underpin this claim, we measured the maximum normalized effective line tension, T^*^/(2*πRγ*_LV_), for the fast and slowly evaporating droplets, which were 7.8 and 2.2, respectively. We observed an increase in T^*^/(2*πRγ*_LV_) as the contact line velocity increased in both cases, meaning that elastic energy stored in the substrate also increased accordingly.

The energy required for additional stretching of the wetting ridge comes from the change in interfacial energy due to decreasing liquid–vapor surface area. In viscoelastic materials, only a fraction of this energy is restituted to the substrate while the rest is dissipated. For low-viscosity fluids like water on viscoelastic substrates, viscoelastic dissipation in the solid dominates viscous dissipation in the fluid^23^. The temporary increase in the measured T^*^/(2*πRγ*_LV_) and the elastic energy stored in the substrate (see above Discussion) signifies that the rate of strain (*dε*/*dt*) imposed through evaporation and contact line motion (∝*dR*/*dt*) is relatively fast compared to the natural substrate relaxation rate (*γ*_LV_/(*Eτ*)), which is determined by viscoelastic effects. These findings support the “quasi-static ridge” assumption to explain the observed wetting behavior.

The mechanism responsible for this dynamically triggered wetting transition can be summarized as follows. For drying droplets with receding contact line velocities of comparable magnitude to the characteristic relaxation rate of the compliant substrate, we observe a sudden reduction in the apparent receding contact angle. Simultaneously, we observe a stretching of the wetting ridge. An asymmetric, strained wetting ridge can occur when the rate of work done on the substrate (storage) through changing droplet surface energy due to evaporation exceeds that of the rate of dissipation in the substrate (loss). We observe that during this wetting transition, the wetting ridge apex starts to tilt inwards toward the droplet. Together, this sharp inward bending apex and the slow responding substrate are responsible for slowing down the contact line motion (also see Fig. 1e) and significantly reducing the apparent receding contact angle necessary to cause depinning, leading to the observed wetting transition.

Our 4D cTFM method enabled us to address and understand the wetting transition, a dynamic wetting phenomenon on a soft substrate. It also allowed us to observe and quantify rapid mesoscopic deformations and relate these to observed macroscale wetting behavior. It had significant advantages over other techniques due to its inherent high temporal and spatial resolution and ability to resolve surface stresses (see Supplementary Table 1 and Supplementary Fig. 13 for a plot showing the limits in substrate stiffness for resolving the wetting ridge profile generated from a water droplet). Previously, droplet wetting on soft materials was characterized with techniques having high-spatial resolution but lacked temporal resolution^30^ or involved X-ray imaging, which is restricted to a very small region and is thus not suitable for observing dynamic wetting phenomena like that investigated here^44^. With 4D cTFM, it is possible to verify or further investigate many recently discovered phenomena in the field, such as stick-slip motion^42,46,47^, droplet durotaxis^26^, or the inverted Cheerios effect^27^. Moreover, the technique allows for studies on collective droplet behavior and multidroplet interaction in general, such as condensation^28^, multidroplet evaporation or freezing^48^ on soft substrates.

## Discussion

In this work, we found that droplets undergo a dynamically triggered wetting transition when drying on a soft material in the presence of the combined effects of substrate viscoelasticity and evaporation rate. By employing 4D cTFM and theoretical considerations, we were able to quantify and explain the underlying mechanism. The discovered phenomenon is expected to have broad significance for additive manufacturing techniques, explaining how residual footprints of droplets can be dependent on evaporation rate and thus possibly setting fabrication speed and resolution limits and giving insight into how droplets are retained on soft materials, which is important for soft membranes or surfaces that are designed to enhance water collection.

## Methods

### Sample preparation and material characterization

We used a polydimethylsiloxane, PDMS Sylgard 184 from Dow Corning (in the following abbreviated PDMS), which we mixed in w/w ratios of 9:1, 30:1, or 50:1 (prepolymer:curing agent). We degassed the silicone elastomer for approximately 40 min. After spin coating at 3000 rpm for 1 min onto a circular glass slide (diameter = 24 mm, thickness = 0.17 mm), the samples were cured in the oven at 90 °C for 35 min. The thickness of the coating was *h* = 30 μm.

We also used Silicone CY 52–276 from Dow Corning (in the following abbreviated Silicone CY). The two components of the silicone and 0.05% (v/v) poly(dimethylsiloxane-b-ethylene oxide) (Polysciences) were mixed thoroughly at a ratio of A:B of 5:6 or 9:10 for 5 min, degassed for 2 min and spin-coated onto a 170 μm thick circular glass slide (diameter = 24 mm, thickness = 0.17 mm) for 1 min at 1500 rpm to achieve a thickness of *h* = 35 µm. The silicone was then cured at 70 °C for 30 min.

The mechanical properties of PDMS 9:1, PDMS 30:1, Silicone CY 5:6 and Silicone CY 9:10 were determined using a tensile testing setup mounted on a MTS 793 testing rig (MTS Systems, Eden Prairie, USA). The elastic moduli of the substrates were measured via an uni-axial tensile strength test (see Supplementary Figs. 4 and 5). The elastic moduli of PDMS 50:1 and Silicone CY 9:10 were taken from literature (ref. ^20^) and from a previous paper (ref. ^35^).

The relaxation behavior of the materials was evaluated using a generalized Maxwell model with one spring and two Maxwell elements (all in parallel) corresponding to two relaxation times—which are the times required for the stress to relax to a constant value when the material is subjected to a constant strain—and was appropriate to capture the behavior of our materials (also see Supplementary Note 2: Considerations for Appropriate Material Model Selection). We determined these relaxation times experimentally by performing step-strain tests where we set the strain (*ε*_0_) to 0.85 for the softer samples (Silicone CY 5:6 and 9:10) and to 0.4 for the harder samples (PDMS 9:1 and 30:1); we repeated the test *N* times. We fitted the generalized Maxwell model equation

2 $$\left| {{\mathbf{\upsigma }}(t)} \right| = E_\infty + E_1{\mathrm{exp}}( - t/\tau _1) + E_2{\mathrm{exp}}( - t/\tau _2){,}$$

to the data where |**σ**(*t*)| is the measured stress, *E* is the elastic modulus, and *τ* is the relaxation time. The subscripts correspond to the Maxwell elements (1 and 2) and the spring (infinity). In this manner we obtained the relaxation times (see Supplementary Figs. 6–9). The mechanism of relaxation is attributed to large molecules sliding past one another or rearranging while maintaining the same network connections. When we calculated the approximate substrate relaxation rate, we used a simplifying case of only one Maxwell element, whose properties better matched the experimentally measured material behavior for the timescales relevant to our experiment, which is the shorter time scale (note: the longer relaxation times of the four materials were in the range between 140 and 1040 s, this is longer than the typical time for droplet evaporation at *rH* = 15%). Thus, *τ* = *τ*_1_ with *τ*_1_ < *τ*_2_.

After preparation, the substrates were kept at all stages in a clean, dust-free and dry environment to prevent fouling. With respect to the ageing properties of dimethyl siloxanes, we always used samples of similar age for experiments (2–3 weeks for Silicone CY and 1–3 weeks for PDMS samples).

The samples that were used for traction force microscopy measurements were subsequently treated in the following way. The QDs were deposited on the substrate by electrohydrodynamic NanoDrip-printing, as reported previously^40,41^. First, the substrate is placed on a conducting grounded plate. A gold-coated glass capillary with an opening diameter of 1–1.5 µm is filled with the colloidal QD solution and brought within 5–10 µm of the substrate using a piezoelectric stage with nanometer precision. By applying voltage pulses between the nozzle and the grounded plate, nanoscale droplets with a diameter of 150–250 nm^35^ are rapidly ejected with frequencies of 100–200 Hz from the apex of a larger meniscus formed at the nozzle exit. The droplets land softly on the substrate (no splashing or sizable spreading beyond the deposited droplet diameter) and the tetradecane evaporates before the arrival of the next droplet, leaving behind only the nanoparticle content. To print one nanodisc of the triangular array, DC voltages of 240–260 V are applied for 120 ms. In this manner, the QDs of several nanodroplets land at the same location each time and form collectively one brightly emitting disc at a well-defined position. Arbitrary patterns are created moving the substrate with a piezoelectric stage. Voltage, pulse length, and stage position are controlled using a custom-built control unit. The red core–shell–shell CdSe–CdS–ZnS QDs with an emission peak at 627 nm were synthesized following a published recipe^49^.

### Macroscopic evaporation experiments

With the goal of evaluating the macroscopic evaporation behavior on compliant substrates in contrast to rigid ones, we made use of an experimental chamber that allowed us to vary the relative humidity within. A schematic of the experimental setup is shown in Fig. 2a. In order to provide the same initial conditions for all the experiments, we needed to ensure that the initial volumes of the droplets were as close as possible. To do so, using the advancing contact angle and assuming a spherical cap, we calculated the required initial contact radius *R*_0_ (hence the volume), then the droplets were dispensed by a tapered and hydrophobically treated glass pipette with an opening of 25–50 µm and inflated using a syringe pump (New Era Pump Systems Inc.) until the desired contact radius was reached. The evaporation process of the droplets, which were illuminated from the back, was captured by a CMOS camera (DCC1645C, Thorlabs) that was placed nearly horizontal (~5°) with a frame rate of 10 s^−1^. The humidity was controlled by means of two valves, which were leading nitrogen gas either through a bubbler to the chamber or directly to the chamber. By adjusting the flow rate of these two valves, the relative humidity in the chamber could be kept constant at a predefined value. The humidity was measured with a LinPicco^™^ Axxx Basic sensor (±3%, Innovative Sensor Technology) and the temperature with an RTD sensor (platinum sensor, Innovative Sensor Technology).

### Traction force microscopy experiments of droplets drying

For measurements of mesoscale deformations at the contact line region and the associated traction forces we used a silicone elastomer (Dow corning, Silicone CY 52–276, ratio 5:6 and 9:10, the latter was already well characterized for the employed method^35^). The samples, which were overprinted with regular arrays of QD discs as discussed earlier, were placed in the above described experimental chamber, except that this time, we implemented an inverted microscope from below (see (a)). Again, the relative humidity was adapted to 15 and 90% in order to observe fast or slow droplet drying and experiments were done at room temperature (*T* = 24.1 ± 0.5 °C). Droplets were dispensed as described above on the as printed arrays. During droplet evaporation, we imaged the mesoscopic surface deformations at the contact line, while simultaneously imaging the droplet from the side. We used ImageJ to measure the contact radius (*R*) and contact angle (θ^*^) in images from the side view. By using a home-built fluorescence microscope we excited the QD discs with a blue laser (wavelength 405 nm) and captured the emitted red light on a sCMOS camera (Andor, Zyla 4.2). To obtain transient 3D data, we recorded *z*-stacks of images at several time steps. The piezo stage (Aerotech, QF-46-100Z-C), upon which the objective was mounted, was translated in the *z*-direction from over a distance of 12–20 µm in steps of 0.5 to 1 µm. At each position an image was acquired (exposure time 20–50 ms), resulting in a scan duration of 1.75 s for the slow evaporating droplets and 1.5 s for fast evaporating droplets.

### Reference-free cTFM algorithm

First, we computed the maximum intensity of the acquired *z*-stack (using ImageJ). The detection of the QD nanodiscs in the images obtained was performed in MATLAB, as described previously^35^ utilizing an adapted version of the cTFM. In brief, local maxima in the image were determined, after applying a Gaussian filter to reduce noise. The exact position of each QD nanodisc was determined by fitting a 2D Gaussian at the position of the local maxima in the original image. Points that were missed by automated detection were added manually. The *z* coordinates of the QD discs were determined by a commercial software (Bitplane Imaris), whereas the *z* coordinates, which were not detected by the Imaris software, were interpolated from the neighboring points.

For the reconstruction of the original QD nanodisc positions on the undeformed substrate the nanodiscs were abstracted as vertices in a mesh. We ran an optimization process to determine the correct connectivity of this mesh in the deformed state. Once the connectivity was determined, the mesh was relaxed such that the vertices were moved to their original positions. The difference between the deformed and the undeformed vertex positions, i.e., the displacements of the QD nanodiscs, were used in the FEA to calculate the forces acting on the substrate. For the FEA we used the commercial finite element code (Abaqus, Dassault Systèmes) together with a hyperelastic material model (Ogden), see ref. ^35^, Supplementary Note 1: Reference-free cTFM Measurement and Supplementary Fig. 2.

### Radial and vertical displacement and effective line tension

In order to obtain radial and vertical displacements with respect to the coordinate system of the droplet, we fitted a circle segment to the QD discs on the contact line, by manually selecting points on the contact line, which were in the field of view (opening angle of circle segment, *α*, see Supplementary Fig. 12). In order to obtain radial and vertical displacements we conducted a coordinate transformation to a cylindrical coordinate system with the origin at the center of the fitted droplet position. We merged the data of *U*_*r*_ and *U*_*z*_ onto one *rz*-plane to obtain wetting ridge shapes. We then fitted a polynomial of degree 5 to *U*_*r*_ and *U*_*z*_, and from this we obtained the displacement values at the contact line, *U*_*r*_(*r* = *R*_+_) and *U*_*z*_(*r* = *R*). The effective line tension, T^*^, which was used for calculation of the elastic energy in the substrate, was computed by integration of tractions over an area *A* from *r* = *R* − *γ*_LV_/*E* to *r* = *R* + *γ*_LV_/*E* with the opening angle *α*. This means that we computed the sum of the products of the stress vector magnitude in the *i*th element in the triangular FEA mesh, |**σ**(**n**)|_*i*_, with the area of the *i*th triangle, *A*_*i*_, whereas the index *i* denotes that the elements are within *A*.

3 $$\begin{array}{*{20}{c}} {T^ \ast \simeq \frac{{2\pi }}{\alpha }\mathop {\sum}\limits_i {\left| {{\mathbf{\upsigma }}({\mathbf{n}})} \right|_iA_i} ,} & {A_i \in A} \end{array}{.}$$

## Supplementary information

Supplementary Information

Transient surface deformation field of a drying droplet

source data

## Data Availability

Figure source data are provided for the following figures: Figs. 1d–f, 2c–f, 3c, f–i, 4, 5b–d, 6c–f, Supplementary Figs. 1, 4–8, 10, and 11c, Any additional dataset generated and/or analyzed during the current study are available from the corresponding author on reasonable request.

## Code availability

Code used in the context of cTFM during the current study is available from the corresponding author on reasonable request.
