# Supplementary material for: Wetting transitions in droplet drying on soft materials
Source: Nat Commun. 2019 Oct 21;10:4776. doi: 10.1038/s41467-019-12093-w (PMC6803709; doi:10.1038/s41467-019-12093-w)
Supplement: Supplementary file 1 — Supplementary Information [file 41467_2019_12093_MOESM1_ESM.pdf]

Supplementary Information

Wetting transitions in droplet drying on soft materials

Julia Gerber, Tobias Lendenmann, Hadi Eghlidi, Thomas M. Schutzius, Dimos Poulikakos**

Laboratory of Thermodynamics in Emerging Technologies, Department of Mechanical and Process Engineering, ETH Zurich, Sonneggstrasse 3, CH-8092 Zurich, Switzerland

* To whom correspondence should be addressed.

Prof. Dimos Poulikakos
ETH Zurich
Laboratory of Thermodynamics in Emerging Technologies
Sonneggstrasse 3, ML J 36
CH-8092 Zürich
SWITZERLAND
Phone: +41 44 632 27 38
Fax: +41 44 632 11 76
dpoulikakos@ethz.ch

Dr. Thomas M. Schutzius
ETH Zurich
Laboratory of Thermodynamics in Emerging Technologies
Sonneggstrasse 3, ML J 38
CH-8092 Zürich
SWITZERLAND
Phone: +41 44 632 46 04
thomschu@ethz.ch

Supplementary Figures

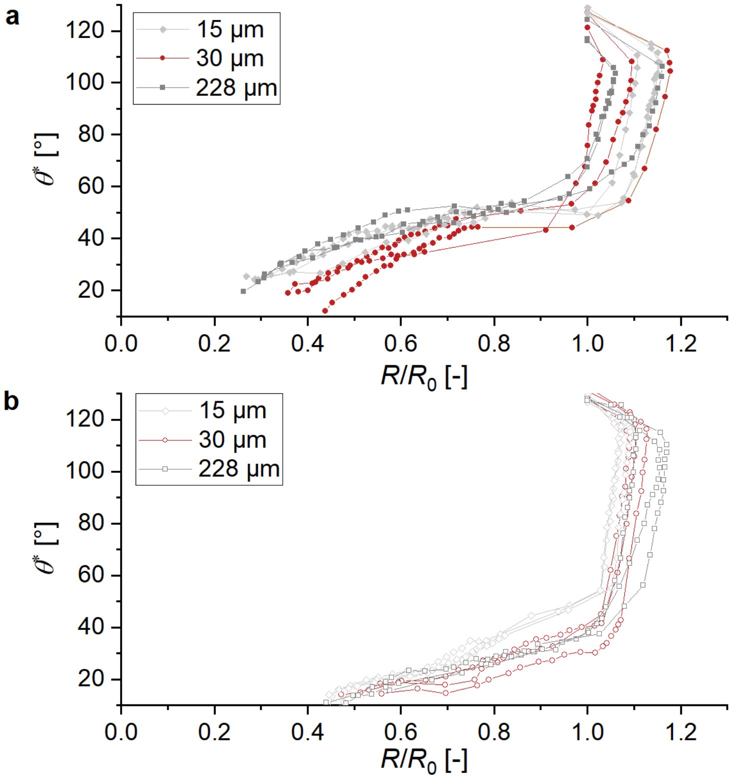

Supplementary Figure 1: Influence of film thickness on receding behavior during droplet evaporation on an elastomer film coated on a glass slide (PDMS Sylgard 184 50:1) at **a**, rH = 89.1 +/- 1.6% and **b**, rH = 16.6 +/-1.0%. Source data are provided as a Source Data file.

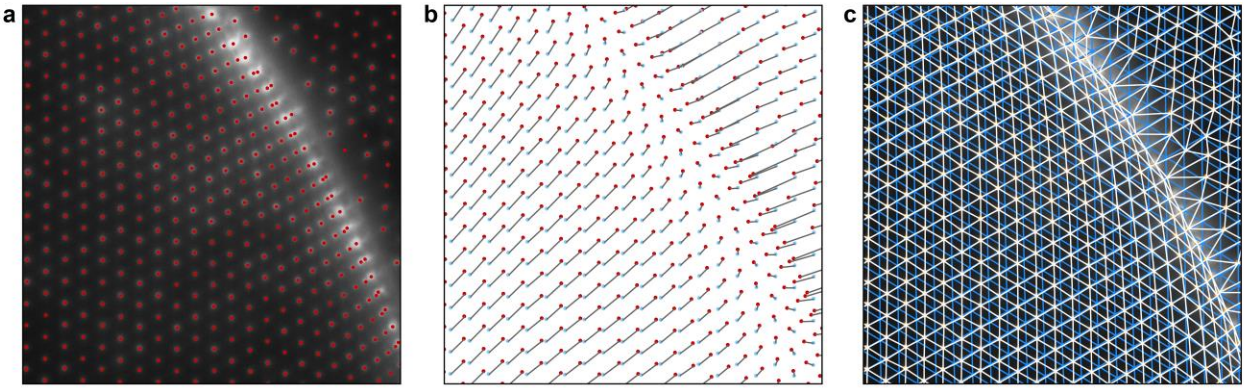

Supplementary Figure 2: Schematic of reference-free cTFM algorithm. **a**, Detected QD discs represented at their fitted centroid through red points. **b**, Globally optimal matching between vertices in a perfect array in blue and the vertices of a detected and deformed array in red. **c**, The perfect array edges (blue) are applied to the matched, deformed vertices to determine the correct connectivity in the deformed array (white edges).

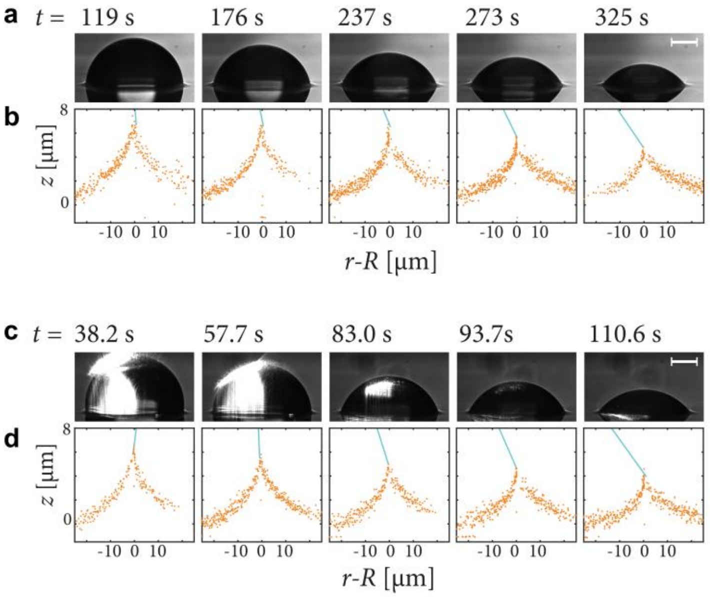

Supplementary Figure 3: Characterizing the effects of humidity and compliance on droplet wetting with 4D reference-free confocal traction force microscopy. Synchronized time series showing the, **a**, macroscopic side-view of a droplet slowly evaporating (rH = 81.2%, V_{placed} = 157 nL) from a compliant solid (Silicone CY 52-276 with a ratio A:B of 5:6) and, **b**, mesoscopic shape of the wetting ridge. Synchronized time series showing the, **c**, macroscopic side-view of a droplet quickly evaporating (rH = 18.7%, V_{placed} = 182 nL) from a compliant solid (Silicone CY 52-276 with a ratio A:B of 5:6) and, **d**, mesoscopic shape of the wetting. Scale bar: **a,** 200 µm, **c,** 200 µm.

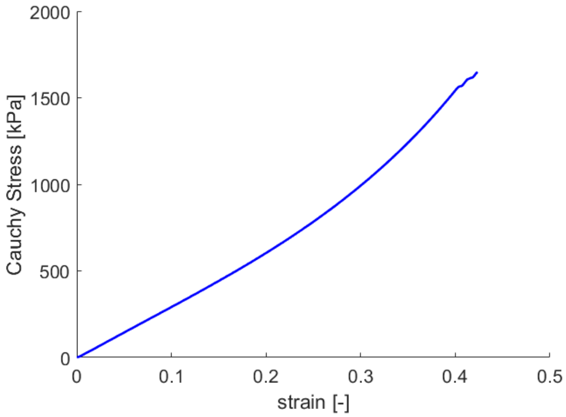

Supplementary Figure 4: Uniaxial tensile strength measurement of PDMS Sylgard 184 9:1. We determined the elastic modulus by linear regression of the data up to 5% strain. E = 2937 kPa. N = 5. Error bars are included, but they are so small that they are not visible. Source data are provided as a Source Data file.

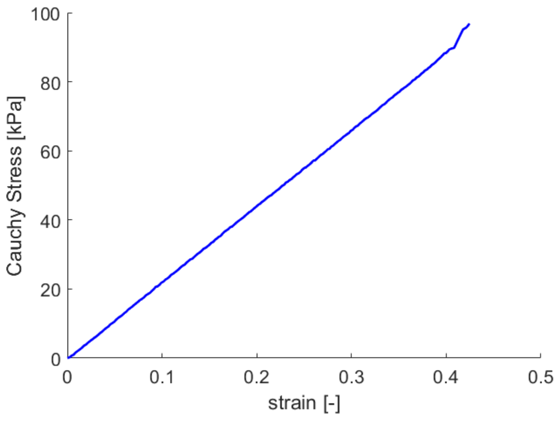

Supplementary Figure 5: Uniaxial tensile strength measurement of PDMS Sylgard 184 30:1. We determined the elastic modulus by linear regression of the data up to 5% strain. E = 218 kPa. N = 5. Error bars are included, but they are so small that they are not visible. Source data are provided as a Source Data file.

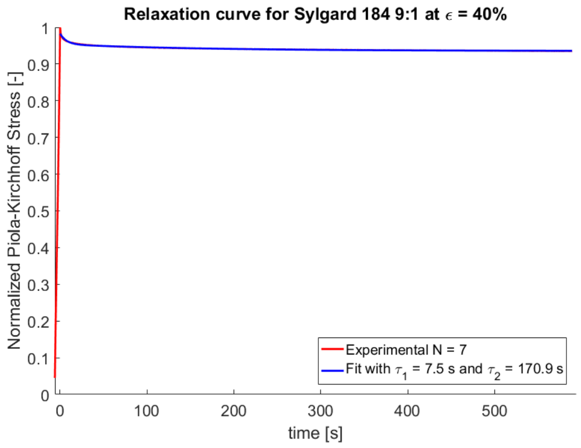

Supplementary Figure 6: Relaxation curve of PDMS Sylgard 184 9:1 at $\varepsilon = 40\%$, N = 7. By fitting a generalized Maxwell solid with two elements we obtained τ_1 =7.5 s and τ_2 =170.9 s. Error bars are included, but they are so small that they are not visible. Source data are provided as a Source Data file.

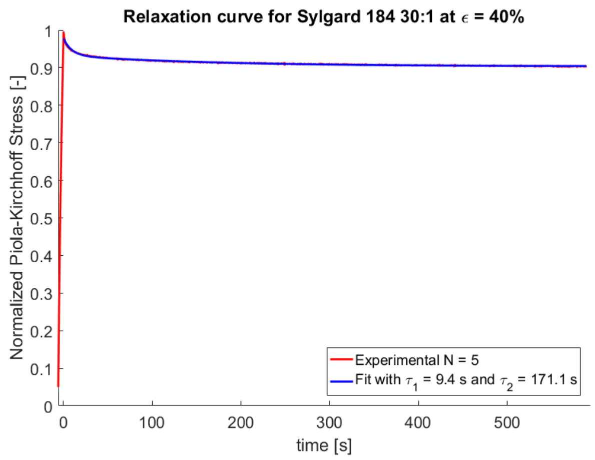

Supplementary Figure 7: Relaxation curve of PDMS Sylgard 184 30:1 at $\varepsilon = 40\%$, N = 5. By fitting a generalized Maxwell solid with two elements we obtained τ_1 =9.4 s and τ_2 =171.1 s. Error bars are included, but they are so small that they are not visible. Source data are provided as a Source Data file.

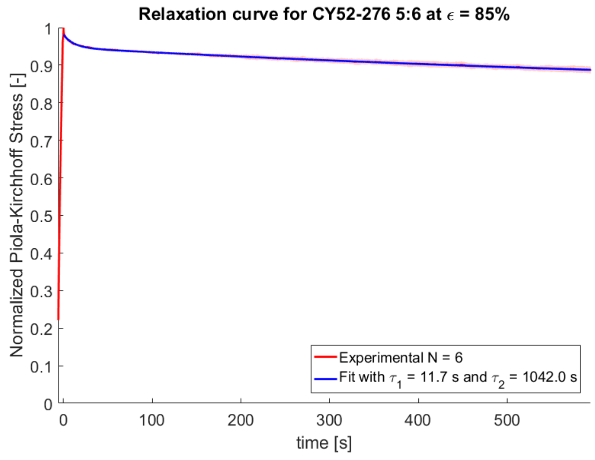

Supplementary Figure 8: Relaxation curve of Silicone CY 52-276 in the ratio of A:B = 5:6 at $\varepsilon = 85\%$, N = 6. By fitting a generalized Maxwell solid with two elements we obtained τ_1 =11.7 s and τ_2 =1042.0 s. Source data are provided as a Source Data file.

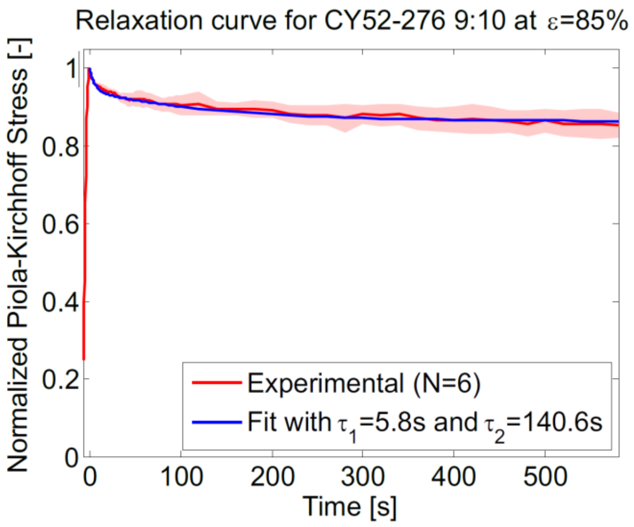

Supplementary Figure 9: Relaxation curve of Silicone CY 52-276 in the ratio of A:B = 9:10 at $\varepsilon = 85\%$, $N = 6$. By fitting a generalized Maxwell solid with two elements we obtained τ_1 =5.8 s and $\tau_2 = 140.6$ s.

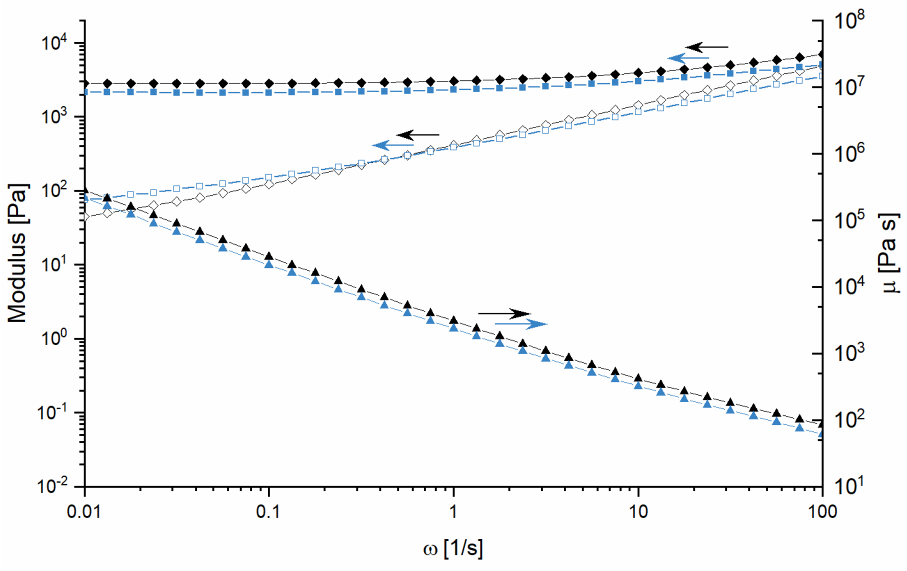

Supplementary Figure 10: Rheological measurement of G'(filled diamond) and G''(empty diamond) and μ (filled triangle) as a function of angular frequency, ω, for PDMS Sylgard 184 50:1 (blue) and Silicone CY 52-276 9:10 (black). The measurement was obtained with a rheometer by Anton Paar (MCR 502). The strain amplitude was 1%. Source data are provided as a Source Data file.

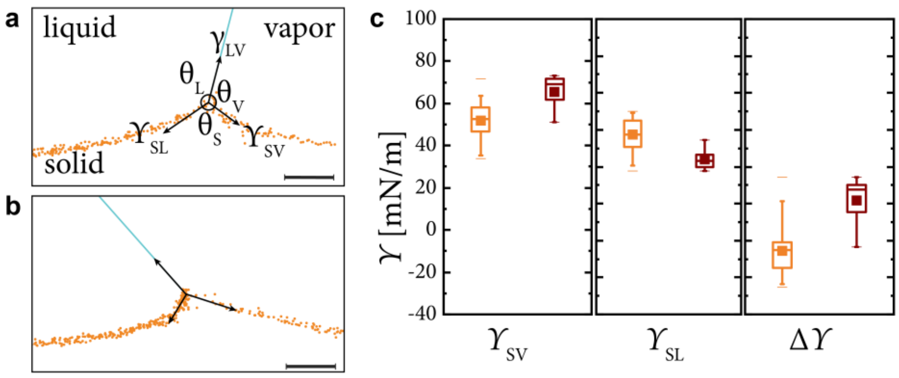

Supplementary Figure 11: Force balance at contact line and solid surface tensions. **a**, Force balance at a static wetting ridge ($|dR/dt| < 10^{-2}$ µm s^{-1}) for a water droplet on Silicone CY 52-276 9:10 between liquid surface tension γ_{LV} and solid surface tensions, Υ_{SV} and Υ_{SL}. Definition of the opening angles of the liquid, vapor, and solid phase, angles θ_L, θ_V, and θ_S, respectively. **b**, Force balance at a receding wetting ridge (with $|dR/dt| = 0.4$ µm s^{-1}). **c**, Υ_{SV}, Υ_{SL}, and $\Delta\Upsilon = \Upsilon_{SV} - \Upsilon_{SL}$ for $|dR/dt| < \gamma_{LV}/E\tau$ (orange, N = 27) and $|dR/dt| > \gamma_{LV}/E\tau$ (red, N = 6). Scale bars: **a**, **b** 5 µm. Source data are provided as a Source Data file for **c**.

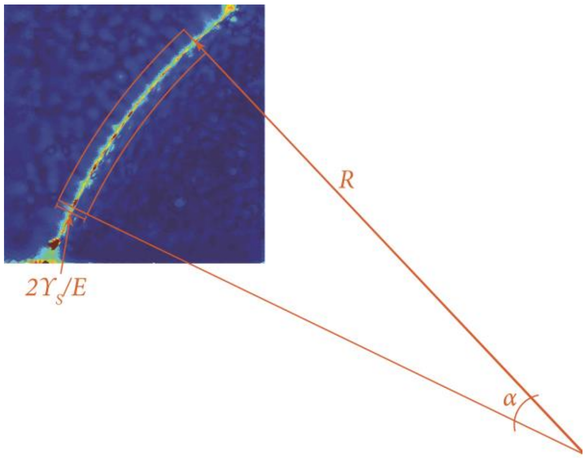

Supplementary Figure 12: Illustration of the parameters used to integrate the traction forces to calculate T^*. Within a circle segment (with the opening angle α) we integrated from $r = R - \Upsilon_S / E$ to $r = R + \Upsilon_S / E$.

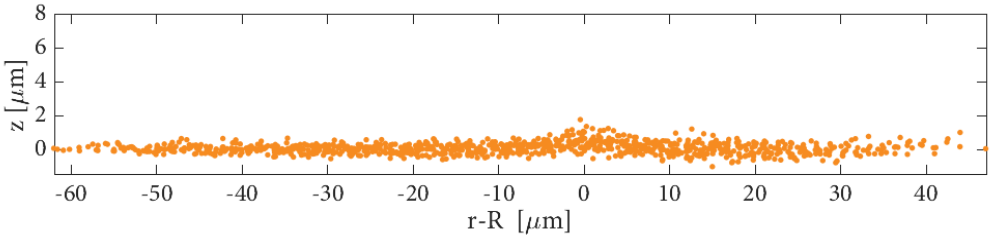

Supplementary Figure 13: Limits of wetting ridge detection with 4D reference-free traction force microscopy. Wetting ridge of a droplet on PDMS Sylgard 184 30:1 (E = 218 kPa).

Supplementary Tables

Supplementary Table 1: Comparison of various wetting ridge imaging techniques according to spatial and temporal resolution and the possibility to compute elastic energy.

Reference	*Spatial resolution*	*Temporal resolution*	*Computation of elastic energy*	*other*
WLI	0.05 µm	~20 s	Not possible, no information about U_r	Not able to resolve surface deformation below droplet
Fluorescence Ref. [5]	distance tracers: random	5 min (combined various time steps into one ridge profile)	Data for U_r and U_z are available, no model for elastic energy stored.	Reference image taken after 45 min (in relaxed state)
X-ray Ref. [6]	0.05 µm	0.05 s	Not possible, no information about U_r	Restricted to small area (given by size of focused X-ray beam), no suited for moving contact line
this paper	0.17 µm pixel^{-1} distance tracers : controllable	~2 s Possibility to tune brightness by printing more QD's into one disc, enables higher temporal resolution	done	Free to move in-plane, observation at different locations possible

Supplementary Note 1: Reference-free cTFM measurement

Meshing

The detected QD nanodiscs (Supplementary Figure 2a) in a regular array are the vertices of a connected graph, where each vertex, apart from the ones along the edge, have six edges that connect them to neighboring vertices. The connectivity for a regular and undeformed array is found using a Delaunay triangulation. For a deformed array, however, the connectivity is no longer trivial and the Delaunay triangulation or simply creating edges to the six closest vertices will create an incorrect grid. To determine the correct connectivity of the deformed array we developed the following procedure. First, we overlay the center of mass of a deformed array and a perfect array and line up the two arrays as well as possible using an iterative closest point (ICP) algorithm. Due to the translation and rotational symmetry of the triangular arrays, ICP is first applied to a small set of vertices around the center of mass and subsequently to vertices on the edge of the array. After the two arrays are aligned, the correspondences between the two arrays are determined such that each vertex in the perfect array is matched with exactly one vertex in the deformed array and vice versa. This matching satisfies a global optimum that minimizes the sum of distances to the power of 10. After the matching is available, the edges of the perfect array are applied to the deformed array such that the connectivity of the deformed array is correct (Supplementary Figure 2b).

Relaxation

After determining the connectivity of the deformed array, it can be relaxed by solving the equation for a regular grid,

$$\mathbf{L} \cdot \mathbf{x} = 0 \ , \tag{1}$$

where **L** is the uniform graph Laplacian and **x** is the vertex coordinates,

$$L_{mn} = \begin{cases} -\sum_{n} L_{mn} & m = n \\ 1 & \text{m is a neighbor of n} \\ 0 & \text{otherwise} \end{cases} . \tag{2}$$

We add as constraints that the vertices on the edge of the array $\mathbf{x}_b$, i.e. the ones that do not have six neighbors, are fixed and do not move. The inner vertices $\mathbf{x}_i$ are left free to move and thereby completely relax the mesh while moving to their original position. The order of the vertices is adjusted and eq. (1) rewritten as,

$$\mathbf{x} = \begin{bmatrix} \mathbf{x}_i \\ \mathbf{x}_b \end{bmatrix} \quad \mathbf{L} = \begin{bmatrix} \mathbf{L}_{ii} & \mathbf{L}_{ib} \\ \mathbf{L}_{bi} & \mathbf{L}_{bb} \end{bmatrix} . \tag{3}$$

Substituting the known $\mathbf{x}_b$ into eq. (1) and solving for $\mathbf{x}_i$ gives us the original positions of the vertices,

$$\begin{bmatrix} \mathbf{L}_{ii} & \mathbf{L}_{ib} \end{bmatrix} \begin{bmatrix} \mathbf{x}_i \\ \mathbf{x}_b \end{bmatrix} = 0 , \tag{4}$$

$$\mathbf{x}_i = -\mathbf{L}_{ii}^{-1} \mathbf{L}_{ib} \mathbf{x}_b . \tag{5}$$

Supplementary Figure 2c shows the relaxed grid compared to the detected grid.

Ogden Model

The material constitutive relations are given by the Ogden model,[1] which describes the material in terms of stain energy potential, W, which defines the strain energy stored in the material per unit of reference volume (volume in the initial configuration) as a function of the strain at that point in the material,

$$W = \sum_{j=1}^{n} \frac{2\mu_j}{\alpha_j^2} \left(\lambda_1^{\alpha_j} + \lambda_2^{\alpha_j} + \lambda_3^{\alpha_j} - 3 \right), \tag{6}$$

where *n* is an integer and μ_j and α_j are both material parameters and λ_i are the principal stretches. The principal stretches are related to the principal nominal strains, ε_i, by $\lambda_i = 1 + \varepsilon_i$. The temperature dependence of W is neglected because experiments were conducted at the same temperature as the measurement of material parameters. The parameters μ_j are linked to the material's bulk modulus by

$$\mu = \sum_{i=1}^{N} \mu_j \ . \tag{7}$$

Then the three principal values of the Cauchy stress, σ_i, can be computed by

$$\sigma_i = \lambda_i \frac{\partial W}{\partial \lambda_i} - p, \tag{8}$$

where *p* is an arbitrary hydrostatic pressure introduced because of the incompressibility constraint.

Supplementary Note 2: Considerations for Appropriate Material Model Selection

We have also tested whether the relaxation time of the materials used here can be defined via the cross over frequency between storage modulus, *G´*, and loss modulus, *G´´*, at the viscous-to-elastic transition point, where $dG'/d\omega < dG''/d\omega$ for the same ω. Supplementary Figure 10 shows *G´* and *G´´* as a function of ω, for PDMS 50:1 and Silicone CY 9:10. We did not find a viscous-to-elastic transition point for the large range of ω values that we studied. Instead, we

found a rubber-elastic plateau; therefore, we cannot define a relaxation time of these materials with this approach.

Supplementary Note 3: Force balance at contact line and strain dependence considerations

Supplementary Figure 11a and b represent the force balances at the contact line of the slowly evaporating droplet (at $t = 69.8\mathrm{s}$) with an almost static contact line ($dR/dt = 1.2\times10^{-3}$ μm s^{-1}) and fast evaporating droplet (at $t = 77.3\mathrm{s}$, $dR/dt = 1.5$ μm s^{-1}) from Supplementary Figure 11a and b, respectively. For the description of wetting phenomena on compliant solids, it is important to make the distinction between the surface energy, γ_{ij}, which is the work per area needed to change the area of a surface by cleaving, and the surface tension, Υ_{ij}, which is the work per area needed to increase the area of a surface by stretching. For liquids, $\Upsilon_{ij} = \gamma_{ij}$.[2,3] For a solid, Υ_{ij} depends on strain and is described by Shuttleworth's relation. Under the assumption of equilibrium, the interfacial tensions should fulfill Neumann's triangle[4] at the contact line. In this manner, we measured the angles at the wetting ridge, as defined in Supplementary Figure 11a, and calculated the solid surface tensions at all instants via Neumann's law,

$$\Upsilon_{\mathrm{SV}} = \gamma_{\mathrm{LV}} \frac{\left(\cos\theta_{\mathrm{L}} \cos\theta_{\mathrm{S}} - \cos\theta_{\mathrm{V}}\right)}{\sin^2\theta_{\mathrm{S}}}, \quad (9)$$

$$\Upsilon_{\mathrm{SL}} = \gamma_{\mathrm{LV}} \frac{\left(\cos\theta_{\mathrm{V}} \cos\theta_{\mathrm{S}} - \cos\theta_{\mathrm{L}}\right)}{\sin^2\theta_{\mathrm{S}}}, \quad (10)$$

with θ_{L}, θ_{V}, and θ_{S} being defined in Supplementary Figure 11a.

In Supplementary Figure 11c we show the calculated solid surface tensions, Υ_{SV}, Υ_{SL}, and their difference $\Delta\Upsilon = \Upsilon_{\mathrm{SV}} - \Upsilon_{\mathrm{SL}}$, for $|dR/dt| < \gamma_{\mathrm{LV}}/(E\tau)$ and $|dR/dt| > \gamma_{\mathrm{LV}}/(E\tau)$. We observed an increase in calculated Υ_{SV} and a decrease of the calculated Υ_{SL} when $|dR/dt|$ exceeds the characteristic rate of substrate relaxation, $\gamma_{\mathrm{LV}}/(E\tau)$.

In order to compare this to the change in solid surface tension due to Shuttleworth's effect, we calculated the maximum difference in strain at the contact line between the fast evaporation and the slow evaporation case (assuming equilibrium). The maximum difference in strains between fast and slow evaporation at the contact line, $\Delta\varepsilon$, are obtained through the results of the finite element analysis (for Silicone CY 52-276):

$$\Delta\varepsilon = \max(\varepsilon_{\mathrm{fast}}) - \max(\varepsilon_{\mathrm{slow}}) = 3.66 - 3.31 = 0.35$$

The subscript 'slow' and 'fast' for the displacements refer to the two measurements, slow and fast evaporation shown in Figure 3a and d. According to Ref. [3] which directly measured the solid surface stress of the same material, Silicone CY52-276 (but with slightly different mixing ratio, *E* = 3 kPa), if $\Delta\varepsilon = 0.35$, then we expect a change of 44 mN m^{-1} in Υ_{SV}. We expect that the strain dependency is weaker with the mixing ratio used here.

We observed that Υ_{SL} decreases by 16.5 mN m^{-1} and Υ_{SV} increases by 16.6 mN m^{-1} when going from a situation where $|dR/dt| < \gamma_{\mathrm{LV}}/(E\tau)$ to $|dR/dt| > \gamma_{\mathrm{LV}}/(E\tau)$. Even though the strain dependency of the solid surface tension cannot be the origin of the asymmetry (there needs to be an unresponsiveness of the wetting ridge), the large strains at the liquid-vapor interface can definitively lead to an increase in Υ_{SV}, which would in turn make the substrate more hydrophilic and thus would make de-pinning less likely.

Supplementary References

1. Ogden, R. W. Large deformation isotropic elasticity – on the correlation of theory and experiment for incompressible rubberlike solids. *Proc. R. Soc. London. A. Math. Phys. Sci.* **326,** 565 LP-584 (1972).

2. Shuttleworth, R. The Surface Tension of Solids. *Proc. Phys. Soc. Sect. A* **63,** 444 (1950).

3. Xu, Q. *et al.* Direct measurement of strain-dependent solid surface stress. *Nat. Commun.* **8,** 555 (2017).

4. Neumann, F. E. & Wangerin, A. *Vorlesungen über die Theorie der Capillarität, gehalten an der Universität Königsberg von Franz Neumann.* (B. G. Teubner, 1894).

5. Jerison, E. R., Xu, Y., Wilen, L. A. & Dufresne, E. R. Deformation of an Elastic Substrate by a Three-Phase Contact Line. *PRL* **186103,** 1–4 (2011).

6. Park, S. J. *et al.* Visualization of asymmetric wetting ridges on soft solids with X-ray microscopy. *Nat. Commun.* **5,** 4369 (2014).
